# Incremental prognostic value of left atrial strain in apical hypertrophic cardiomyopathy: a cardiovascular magnetic resonance study

**DOI:** 10.1007/s00330-024-11058-y

**Published:** 2024-09-18

**Authors:** Yun Tang, Xuan Ma, Jiaxin Wang, Shujuan Yang, Zhixiang Dong, Xiuyu Chen, Kankan Zhao, Zhuxin Wei, Jing Xu, Yanyan Song, Xiaorui Xiang, Chen Cui, Yanjie Zhu, Kai Yang, Shihua Zhao

**Affiliations:** 1 Department of Magnetic Resonance Imaging, Fuwai Hospital, National Center for Cardiovascular Diseases, State Key Laboratory of Cardiovascular Disease, Chinese Academy of Medical Sciences and Peking Union Medical College, Beijing, China; 2 Research Center for Medical AI, Shenzhen Institutes of Advanced Technology, Chinese Academy of Sciences, Shenzhen, China; 3 Paul C. Lauterbur Research Center for Biomedical Imaging, Shenzhen Institutes of Advanced Technology, Chinese Academy of Sciences, Shenzhen, China

**Keywords:** Apical hypertrophic cardiomyopathy, Magnetic resonance imaging, Atrial function, Prognosis

## Abstract

**Objectives:**

This study aimed to evaluate the prognostic value of left atrial (LA) strain in patients with apical hypertrophic cardiomyopathy (ApHCM), as assessed by cardiac magnetic resonance (CMR) imaging.

**Methods:**

Four hundred and five consecutive patients with ApHCM who underwent CMR examination were retrospectively included. The study endpoint included all-cause death, heart transplant, aborted sudden cardiac death, hospitalization for heart failure, stroke, and new-onset atrial fibrillation (AF).

**Results:**

After a median follow-up of 97 months, 75 patients (18.5%) reached the endpoint. Patients were divided into two groups based on the median LA reservoir strain of 29.4%. The group with lower LA reservoir strain had thicker maximum wall thickness, greater late gadolinium enhancement extent, and smaller end-diastolic volume index, stroke volume index, and cardiac index (all *p* < 0.02). For LA parameters, this subgroup showed greater diameter and volume index and worse ejection fraction, reservoir, conduit, and booster strain (all *p* < 0.001). In the multivariable model, age (HR 1.88, 95% CI: 1.06–3.31, *p* = 0.030), baseline AF (HR 2.95, 95% CI: 1.64–5.28, *p* < 0.001), LA volume index (LAVi) (HR 2.07, 95% CI: 1.21–3.55, *p* = 0.008) and LA reservoir strain (HR 2.82, 95% CI: 1.51–5.26, *p* = 0.001) were all associated with the outcome. Adding LAVi and LA reservoir strain in turn to the multivariable model (age and baseline AF) resulted in significant improvements in model performance (*p* < 0.001).

**Conclusion:**

In ApHCM patients, LA reservoir strain is independently associated with cardiovascular risk events and has an incremental prognostic value.

**Clinical relevance statement:**

Left atrial reservoir strain measured by cardiac magnetic resonance is highly correlated with the prognosis of apical hypertrophic cardiomyopathy and has potential incremental value in the prognosis of major adverse cardiac events.

**Key Points:**

- *Left atrial (LA) strain parameters may be useful for risk stratification and treatment of apical hypertrophic cardiomyopathy (ApHCM).*
- *Apical hypertrophic cardiomyopathy (ApHCM) is independently associated with LA morphology and function.*
- *Cardiac MR examination, especially its feature-tracking technology, provides the possibility to prognosticate ApHCM at an early stage.*

**Graphical Abstract:**

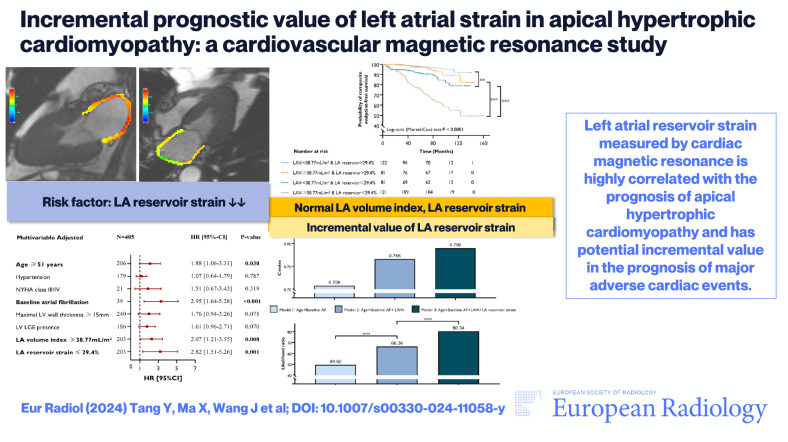

## Introduction

Apical hypertrophic cardiomyopathy (ApHCM), as a subtype of hypertrophic cardiomyopathy (HCM), refers to myocardial hypertrophy involving mainly the left ventricular (LV) apex. The incidence of ApHCM varies greatly in different regions, among which the incidence in Japan is significantly higher than that in Western countries [1, 2]. Due to the insufficient sample size of the cohort, the incidence of all-cause mortality or other major adverse cardiovascular events (MACE) also varied across previous studies [3–5]. The prognosis of HCM has been shown to be correlated with LV outflow tract obstruction, degree of hypertrophy, and late gadolinium enhancement (LGE) in numerous previous studies [6–9]. Diastolic dysfunction is known to be predominant in HCM. Moreover, left atrial (LA) morphology and function may be considered as a diastolic function’s weather vane, which could predict the follow-up in the early stage [10, 11]. Altered LA volume, which represents structural remodeling, has been shown in several studies to be of significant prognostic value [12–14].

Cardiac magnetic resonance (CMR) imaging (MRI) is an excellent tool for detecting and evaluating HCM, and in recent years the use of feature-tracking techniques has allowed mechanical dysfunction to be detected before morphological changes [15, 16]. LA longitudinal strain (including reservoir, conduit, and booster pump function) is correlated with prognosis, as has been demonstrated in previous CMR prognostic studies of HCM to a certain extent [17, 18]. However, there is currently a lack of guidelines for disease surveillance and risk stratification specific to ApHCM, and the number and size of large long-term prognostic studies of ApHCM are limited and do not yet involve LA strain. Therefore, our study aimed to investigate the prognostic value of LA strain in patients with ApHCM.

## Materials and methods

### Study population

This study was performed in accordance with the principles of the Declaration of Helsinki and was approved by the institutional review board of Fuwai Hospital. Written informed consent was waived because of the retrospective analysis of anonymous data with no risk. A total of 467 ApHCM patients at Fuwai Hospital (National Center for Cardiovascular Diseases of China) from June 2009 to December 2014 who were diagnosed or confirmed by CMR were retrospectively included in this study. The diagnosis of ApHCM was based on the CMR diagnostic criteria, which included unexplained LV hypertrophy involving mainly the LV apex below the papillary muscle level, with an apical wall thickness ≥ 15 mm or a ratio of maximum apical to the posterior wall thickness ≥ 1.5 at the end-diastole [19–22]. The exclusion criteria were as follows: significant ischemic heart disease, other cardiac diseases, surgical interventions, poor image quality including those without sinus rhythm (e.g., atrial fibrillation (AF)) during scanning, and missing data. According to different phenotypes of hypertrophy, patients can be divided into “pure” ApHCM and “mixed” ApHCM. “Pure” ApHCM refers to hypertrophy confined to the apical segment below the level of the LV papillary muscle. “Mixed” ApHCM is defined as mainly apical hypertrophy but also has hypertrophy in the middle or base of the ventricular wall [23, 24]. Echocardiographic Doppler assessment was used to detect mid-LV obliteration and to measure pressure gradient [25].

### CMR protocol

The CMR images were acquired as previously described [21, 26]. All scans were performed on a 1.5-T CMR scanner (Magnetom Avanto, Siemens). Standard cine imaging with steady-state free precession was performed, including LV two-chamber, four-chamber, LV outflow tract, and short-axis planes. LGE images were acquired at 10–15 min post-intravenous injection of 0.2 mmol/kg of gadolinium-DTPA (Magnevist, Bayer Healthcare Pharmaceuticals). All patients received routine breath-hold training before the examination.

### CMR analysis

**Fig. 1:**
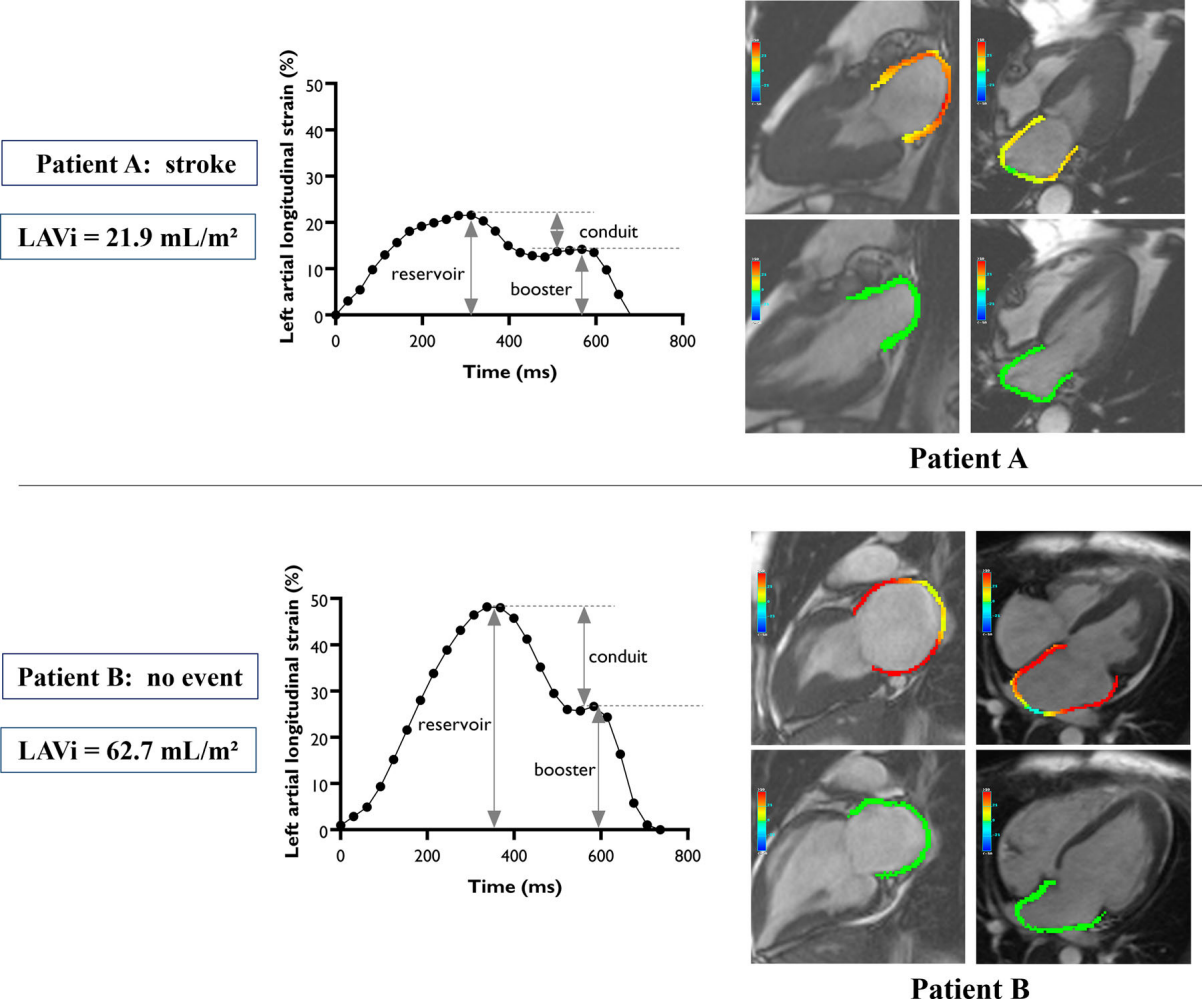
CMR feature-tracking acquired measurements. Patient A, a 61-year-old male patient, was classified as a “mixed” type of ApHCM. He had a normal LAVi of 21.9 mL/m^2^ but significantly abnormal LA reservoir strain, conduit strain, and booster strain of 21.6%, 7.4%, and 14.2%, respectively. 100 months after CMR, patient A had a stroke. Patient B, a 45-year-old female patient, was classified as a “mixed” type of ApHCM. She had a significantly enlarged LA with an LAVi of 62.7 mL/m^2^ but normal LA reservoir strain, conduit strain, and booster strain of 48.2%, 21.5%, and 26.7%, respectively. No MACE occurred in patient B until the end of the 104-month follow-up after CMR scanning. CMR, cardiac magnetic resonance; ApHCM, apical hypertrophic cardiomyopathy; LAVi, left atrial volume index; LA, left atrial; MACE, major adverse cardiac event

The CMR images were analyzed using commercially available software (Circle CVI^42^) by two radiologists (S.Z. and X.C.) with at least 15 years of experience, who were blinded to clinic data and outcomes. Basic CMR parameters were collected from the horizontal and vertical long-axis views according to current guidelines [27]. The contours of the endocardium and epicardium in the left atrium and ventricle were quantified by manually drawing in the end-diastolic and end-systolic phases and simultaneous mitral and apical positioning on two- and four-chamber cine. LA volume was defined as the maximal volume before the mitral valve opening [28]. LA reservoir strain reflected the global peak longitudinal strain during LV systole. LA conduit strain reflected the blood flow from LA to LV and was the global longitudinal strain during LV early diastole. LA booster strain was the global longitudinal strain during LV late diastole [29]. LA peak longitudinal reservoir, conduit, and booster strain were assessed based on LV four-chamber and two-chamber cine sequences using an automated 2-D feature-tracking algorithm [30] (Fig. 1). Similarly, the LGE in the LV was quantified by selecting a remote area of normal myocardium as a region of interest (ROI) in each enhanced short-axis slice and contours were manually adjusted when needed, with a threshold signal intensity of at least 5 standard deviations above the mean of the ROI [21]. The presence of LGE was defined as an over 1% range. The LGE extent refers to a percentage of the total myocardial mass.

To verify the reproducibility of the study, for the intra-observer variability, the LA strain of 20 randomly selected patients was re-measured one month later. For the inter-observer variability, another investigator (K.Y.) measured the parameters blinded to the former data.

### Clinical follow-up

Patient follow-up information was based on subsequent readmissions in the electronic system (*n* = 248) and telephone calls (*n* = 157). Follow-up time was calculated from the first time it was diagnosed or confirmed by CMR. The endpoint was a composite of all-cause death, heart transplant, aborted sudden cardiac death (SCD), hospitalization for heart failure (HF), stroke, and new-onset AF.

### Statistical analysis

Statistical analyses were performed by using SPSS (version 25.0, IBM) and R Studio (version 2022.07.0, PBC). Categorical variables were expressed as numbers and percentages, while continuous variables were expressed as mean ± standard deviation and medians with interquartile range (IQR), respectively, according to normality or not.

All patients were divided into two groups based on the median LA reservoir strain of 29.4%. Group comparisons of categorical variables were analyzed using either the Chi-square test or Fisher’s exact test. Continuous variables were compared using either the Student’s *t*-test or the Kruskal–Wallis test (adjusted by the Bonferroni method), chosen as required. Univariable and multivariable associations of risk factors with MACE were determined by Cox proportional hazards regression analyses and effect sizes were expressed as hazard ratios (HRs) with 95% CIs. Variables with *p*-value < 0.05 in the univariable analysis were included in multivariable models.

In addition, receiver operating characteristic (ROC) curves were utilized to demonstrate the best predictive LA parameters for major cardiovascular risk event endpoints. Patients were grouped based on the median LA volume index (LAVi) and LA reservoir strain and the Kaplan–Meier (KM) survival curves were plotted to analyze their predictive value. A two-tailed *p*-value < 0.05 was considered statistically significant. The effectiveness of the different models was compared using Harrell’s C-index and log-likelihood ratio test. The intra-class correlation coefficient (ICC) analysis was used to assess the inter- and intra-observer variability for LA strain parameters. Interaction tests were performed to assess statistically significant subgroup differences.

## Results

### Baseline characteristics

**Fig. 2:**
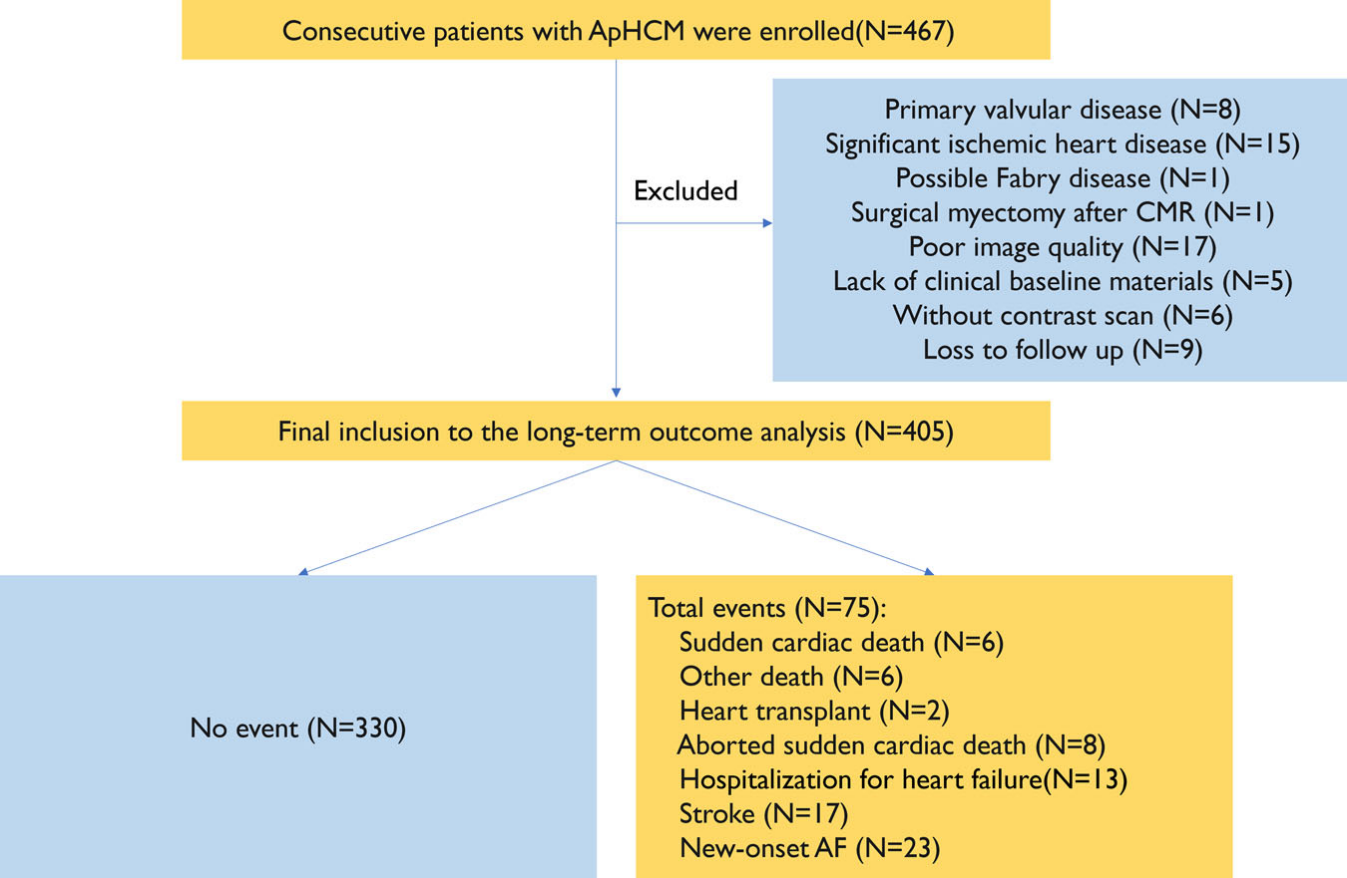
Flow chart of patients enrolled and their follow-up outcomes. ApHCM, apical hypertrophic cardiomyopathy; AF, atrial fibrillation

After application of the exclusion criteria, a total of 405 individuals were included in this study (patients with events *n* = 75; patients with no event *n* = 330), including 243 patients with “pure” type and 162 patients with “mixed” type ApHCM. A flow chart is shown in Fig. 2. Clinical and CMR data were collected for a median follow-up of 97 (75–110) months, yielding an event rate of 18.5%. The clinical and CMR baseline data are presented in Tables 1 and  2, respectively.

**Table 1:** Clinical data divided into subgroups with LA reservoir strain median of 29.4%

	Total (*n* = 405)	LA reservoir strain ≤ median (29.4%) group (*n* = 203)	LA reservoir strain > median (29.4%) group (*n* = 202)	*p*-value
Male, %	328 (81.0)	156 (76.8)	172 (85.1)	**0.033**
Age, years	51.26 ± 13.10	56.87 ± 11.98	45.63 ± 11.71	**<** **0.001**
BMI, kg/m^2^	25.87 ± 3.13	25.88 ± 3.13	25.86 ± 3.13	0.965
Systolic blood pressure, mmHg	117.7 ± 10.9	118.7 ± 10.3	116.7 ± 11.4	0.072
Diastolic blood pressure, mmHg	74.2 ± 7.6	74.7 ± 7.6	73.7 ± 7.5	0.183
NYHA class III/IV,%	21 (5.2)	12 (5.9)	9 (4.5)	0.874
LV Outflow tract obstruction, %	11 (2.7)	9 (4.4)	2 (1.0)	**0.033**
Mitral regurgitation ≥ moderate, %	1 (0.2)	1 (0.5)	0	0.391
Clinical symptoms, %				
Asymptomatic	113 (27.9)	44 (21.7)	69 (34.2)	**0.005**
Chest pain	112 (27.7)	61 (30.0)	51 (25.2)	0.281
Palpitation	99 (24.4)	60 (29.6)	39 (19.3)	**0.016**
Dyspnea	70 (17.3)	40 (19.7)	30 (14.9)	0.197
Syncope	13 (3.2)	9 (4.4)	4 (2.0)	0.162
Amaurosis	6 (1.5)	4 (2.0)	2 (1.0)	0.415
Dizziness	31 (7.7)	19 (9.4)	12 (5.9)	0.197
Fatigue	21 (5.2)	13 (6.4)	8 (4.0)	0.269
Family history of HCM, %	46 (11.4)	27 (13.3)	19 (9.4)	0.218
History of hypertension, %	179 (44.2)	103 (50.7)	76 (37.6)	**0.008**
Medications, %				
Beta blockers	165 (40.7)	87 (42.9)	78 (38.6)	0.385
Calcium channel blockers	60 (14.8)	36 (17.7)	24 (11.9)	0.097
ACEI/ARB	68 (16.8)	38 (18.7)	30 (14.9)	0.298
Aspirin	8 (2.0)	6 (3.0)	2 (1.0)	0.155
Warfarin	11 (2.7)	8 (3.9)	3 (1.5)	0.128
History of electrocardiography, %				
Atrial fibrillation	39 (9.6)	34 (16.7)	5 (2.5)	**<** **0.001**
Non-sustained ventricular tachycardia	2 (0.5)	2 (1.0)	0	0.158

Continuous variables are presented as mean ± SD or median (IQR), and categorical variables are presented as numbers (%)

*LA* left atrial, *BMI* body mass index, *NYHA* New York Heart Association, *LV* left ventricular, *HCM* hypertrophic cardiomyopathy, *ACEI* angiotensin-converting enzyme inhibitor, *ARB* angiotensin receptor blocker

Bold values indicate statistical significance

**Table 2:** CMR data divided into subgroups with LA reservoir strain median of 29.4%

	Total (*n* = 405)	LA reservoir strain ≤ median (29.4%) group (*n* = 203)	LA reservoir strain > median (29.4%) group (*n* = 202)	*p*-value
Left ventricular parameters				
End-diastolic dimension, mm	50 (47, 53)	50.00 (46.00, 53.00)	50.00 (47.75, 53.00)	0.363
Maximal wall thickness, mm	15.00 (13.00, 18.00)	16.00 (14.00, 19.00)	14.00 (12.00, 17.00)	**<** **0.001**
Ejection fraction, %	67.45 ± 7.06	67.48 ± 7.29	67.41 ± 6.84	0.917
End-diastolic volume index, mL/m^2^	68.76 (60.66, 77.31)	65.91 (57.92, 75.66)	70.99 (62.93, 79.24)	**<** **0.001**
End-systolic volume index, mL/m^2^	23.63 (20.75, 28.16)	23.62 (20.47, 27.74)	23.70 (20.93, 28.33)	0.328
Stroke volume index, mL/m^2^	44.07 (38.25, 51.23)	41.55 (36.15, 48.55)	45.84 (40.80, 52.46)	**<** **0.001**
Cardiac index, L/min/m^2^	2.95 (2.49, 3.45)	2.82 (2.32, 3.38)	3.08 (2.68, 3.54)	**<** **0.001**
Mass index, g/m^2^	63.10 (53.63, 75.75)	65.52 (53.27, 80.39)	62.53 (55.13, 71.40)	0.207
LGE presence, %	186 (45.9)	101 (49.8)	85 (42.1)	0.122
LGE extent, %	0 (0, 2.62)	0 (0, 3.95)	0 (0, 2.01)	**0.017**
Apical aneurysm presence, %	6 (1.5)	5 (2.5)	1 (0.5)	0.102
Mid-LV obliteration presence, %	7 (1.7)	6 (3.0)	1 (0.5)	0.057
Left atrial parameters				
Anteroposterior diameter, mm	35.00 (30.00, 39.00)	37.00 (31.00, 42.00)	32.00 (29.00, 36.00)	**<** **0.001**
Volume index, mL/m^2^	38.77 (30.42, 49.31)	43.68 (31.41, 58.13)	36.28 (28.52, 62.65)	**<** **0.001**
Ejection fraction, %	54.20 (46.04, 60.44)	46.46 (38.30, 52.46)	59.53 (56.20, 62.98)	**<** **0.001**
Reservoir strain, %	29.40 (23.00, 36.10)	23.00 (17.30, 26.50)	36.10 (33.00, 40.35)	**<** **0.001**
Conduit strain, %	11.60 (8.10, 16.30)	8.50 (5.90, 10.70)	16.10 (12.98, 19.43)	**<** **0.001**
Booster strain, %	17.40 (13.20, 21.25)	13.40 (9.70, 15.90)	21.05 (18.50, 24.13)	**<** **0.001**

Continuous variables are presented as mean ± SD or median (IQR), and categorical variables are presented as numbers (%)

*CMR* cardiac magnetic resonance, *LA* left atrial, *LGE* late gadolinium enhancement, *LV* left ventricular

Bold values indicate statistical significance

A total of 7 of 405 ApHCM patients had mid-LV obliteration with a Doppler systolic gradient range of 25–96 mmHg. Six of them had LA reservoir strain below the median.

In the subgroup with LA reservoir strain no greater than 29.4%, a higher proportion of men, advanced age, LV outflow tract obstruction, and history of palpitations, hypertension, and AF were observed, while asymptomatic patients were less common (all *p* < 0.04). Regarding the LV CMR data, the subgroup with lower LA reservoir strain showed thicker maximum wall thickness (*p* < 0.001), greater LGE extent (*p* = 0.017), and smaller end-diastolic volume index, stroke volume index, and cardiac index (*p* < 0.001).

In terms of LA parameters, the subgroup with lower LA reservoir strain had a greater LA diameter (median 37.00, IQR: 31.00 to 42.00 mm vs. median 32.00, IQR: 29.00 to 36.00 mm) and volume index (median 43.68, IQR: 31.41 to 58.13 mL/m^2^ vs. median 36.28, IQR: 28.52 to 62.65 mL/m^2^) and worse LA ejection fraction (EF) (median 46.46, IQR: 38.30 to 52.46% vs. median 59.53, IQR: 56.20 to 62.98%), reservoir (median 23.00, IQR: 17.30 to 26.50% vs. median 36.10, IQR: 33.00 to 40.35%), conduit (median 8.50, IQR: 5.90 to 10.70% vs. median 16.10, IQR: 12.98 to 19.43%) and booster strain (median 13.40, IQR: 9.70 to 15.90% vs. median 21.05, IQR: 18.50 to 24.13%) (all *p* < 0.001).

### Factors in predicting endpoint events

The unadjusted-univariable Cox regression analysis in Table S1 revealed that age, history of hypertension, high level of the New York Heart Association (NYHA) functional classification (III/IV), baseline AF history, LV parameters (maximum ventricular wall thickness, LGE), and LA parameters (anteroposterior diameter, volume index, EF, strain) were significantly associated with patient outcome in those with ApHCM. All continuous variables in Table S1 were also converted to categorical variables based on the median, and the results remained statistically significant.

**Fig. 3:**
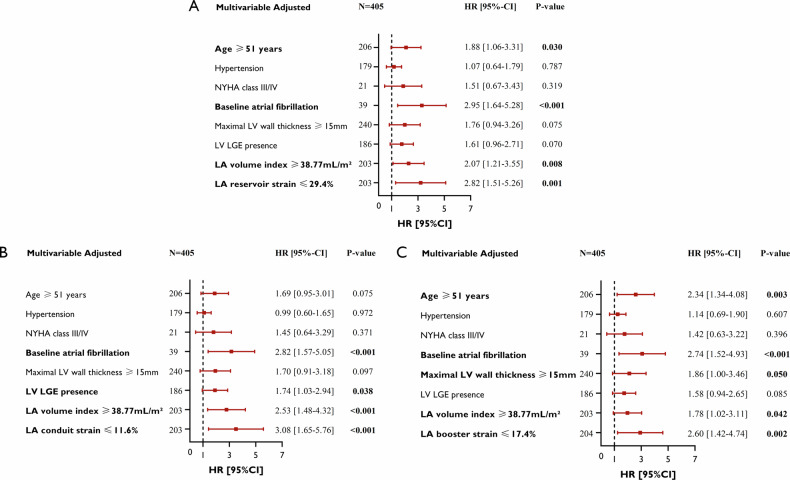
Adjusted-multivariable predictors of cardiovascular endpoint performed with dichotomous form in all patients. LA reservoir strain (**A**), LA conduit strain (**B**), and LA booster strain (**C**) were incorporated separately in the multivariable Cox analysis model due to their interrelatedness. NYHA, New York Heart Association; LV, left ventricular; LA, left atrial; LGE, late gadolinium enhancement; HR, hazard ratio

As four categorical variables (hypertension, NYHA class, baseline AF, and LV LGE presence) would be included in the multivariable analysis and the inevitable collinearity of LA parameters (between LAVi and LA strain), all continuous variables were dichotomized to categorical variables. Also to avoid collinearity, LA three-phase longitudinal strain (including reservoir, conduit, and booster strain) would enter into the multivariable model separately. The results of the multivariable regression analysis are presented in the forest plot in Fig. 3. As shown in Fig. 3A, being over 51 years old (HR 1.88, 95% CI: 1.06–3.31, *p* = 0.030), baseline AF (HR 2.95, 95% CI: 1.64–5.28, *p* < 0.001), LAVi ≥ 38.77 mL/m^2^ (HR 2.07, 95% CI: 1.21–3.55, *p* = 0.008) and LA reservoir strain ≤ 29.4% (HR 2.82, 95% CI: 1.51–5.26, *p* = 0.001) were all independently associated with MACE in patients with ApHCM. Furthermore, when the model incorporated the conduit (Fig. 3B) and booster strain (Fig. 3C), both had independent predictive values. In Table 3, LAVi, LA EF, reservoir strain, conduit strain, and booster strain were respectively included as continuous variables in each model. LAVi, reservoir strain, and booster strain as continuous variables were still independently related to the outcome (*p* ≤ 0.001), while the *p* values of LA EF and conduit strain were > 0.05. As the KM analysis shown in Fig. S1, patients with LGE extent ≥ 1.33% had a higher cumulative incidence of MACE than those with LGE extent < 1.33% (log-rank *p* < 0.0001).

**Table 3:** Multivariable predictors of cardiovascular endpoint performed with continuous form in all patients

Multivariable adjusted	HR (95%-CI)	*p*-value	HR (95%-CI)	*p*-value	HR (95%-CI)	*p*-value	HR (95%-CI)	*p*-value	HR (95%-CI)	*p*-value
Age, years	1.03 (1.01, 1.05)	**0.010**	1.04 (1.01, 1.06)	**0.002**	1.04 (1.02, 1.06)	**<** **0.001**	1.04 (1.02, 1.06)	**<** **0.001**	1.04 (1.02, 1.06)	**<** **0.001**
Hypertension, %	1.03 (0.62, 1.73)	0.909	1.02 (0.61, 1.72)	0.929	1.10 (0.66, 1.83)	0.712	1.12 (0.68, 1.86)	0.659	1.06 (0.63, 1.78)	0.826
NYHA III/IV,%	2.01 (0.90, 4.46)	0.088	2.08 (0.94, 4.61)	0.071	2.03 (0.91, 4.53)	0.082	2.10 (0.95, 4.64)	0.065	1.98 (0.89, 4.40)	0.096
Baseline AF, %	1.71 (0.85, 3.46)	0.135	2.80 (1.51, 5.18)	**0.001**	1.45 (0.69, 3.03)	0.326	2.03 (1.03, 3.99)	**0.040**	2.15 (1.05, 4.41)	**0.036**
LV maximal wall, mm	1.06 (1.00, 1.12)	0.070	1.06 (1.00, 1.13)	**0.044**	1.07 (1.01, 1.13)	**0.029**	1.08 (1.02, 1.15)	**0.005**	1.07 (1.01, 1.14)	**0.014**
LV LGE extent, %	1.05 (1.00, 1.10)	0.077	1.06 (1.00, 1.11)	**0.037**	1.04 (0.99, 1.09)	0.163	1.03 (0.98, 1.09)	0.182	1.04 (0.99, 0.10)	0.105
LAVI, mL/m^2^	-	**-**	**-**	**-**	**-**	**-**	1.02 (1.01, 1.04)	**0.001**	-	**-**
LAEF, %	-	**-**	**-**	**-**	**-**	**-**	-	**-**	0.98 (0.96, 1.00)	> 0.050
LA reservoir strain, %	0.95 (0.92, 0.98)	**0.001**	**-**	**-**	**-**	**-**	**-**	**-**	-	**-**
LA conduit strain, %	-	**-**	0.96 (0.90, 1.02)	0.161	**-**	**-**	**-**	**-**	-	**-**
LA booster strain, %	-	**-**	**-**	**-**	0.92 (0.88, 0.96)	**<** **0.001**	**-**	**-**	-	**-**

*HR* hazard ratio, *NYHA* New York Heart Association, *AF* atrial fibrillation, *LV* left ventricular, *LA* left atrial, *EF* ejection fraction, *LAVi* left atrial volume index

Bold values indicate statistical significance

### Prognostic value of LA structural and functional indicators

**Fig. 4:**
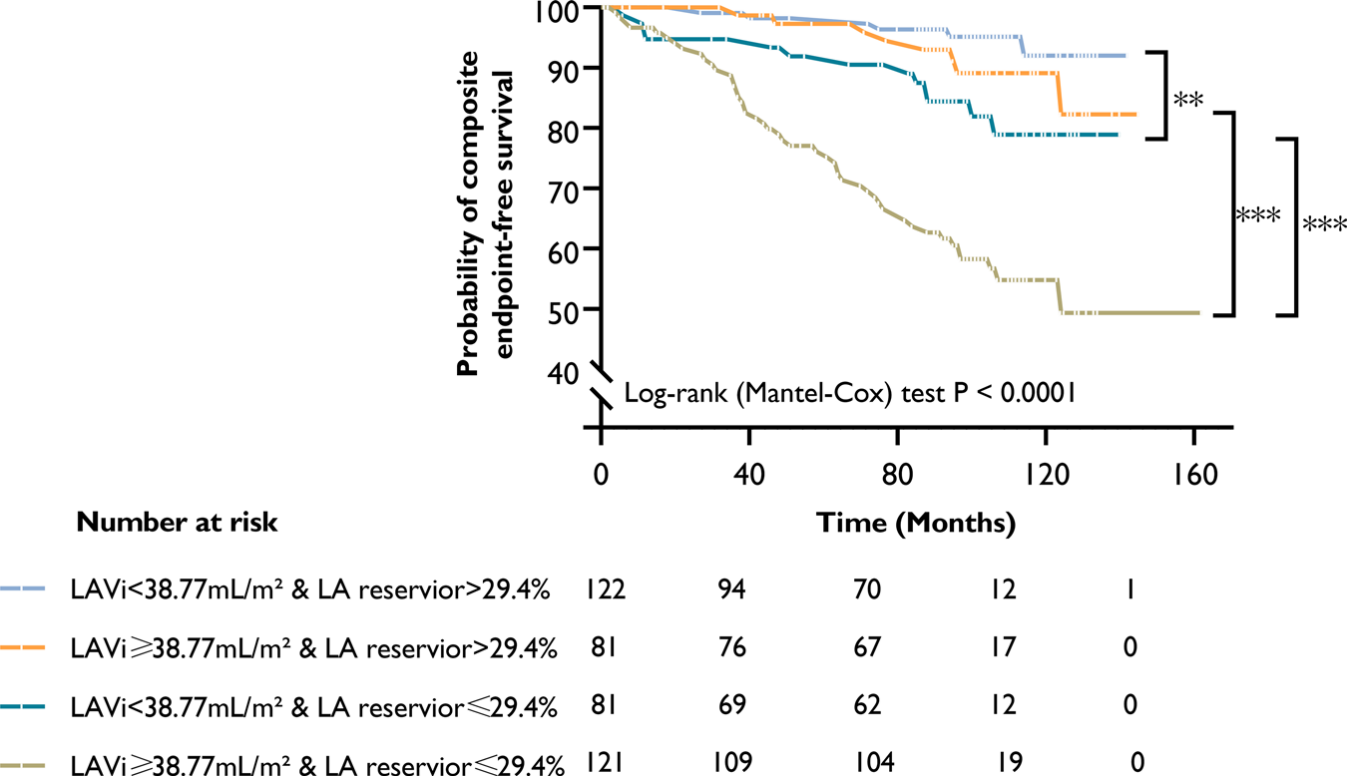
Kaplan–Meier survival curves for subgroups divided by LA reservoir strain and LAVi median. LA, left atrial; LAVi, left atrial volume index. ** indicating *p* < 0.01; *** indicating *p* < 0.001

The ROC curves in Fig. S2 revealed the prognostic value of LAVi (area under the curve (AUC): 0.819) and LA reservoir, conduit, and booster strains (AUC: 0.769, 0.743 and 0.721, respectively). In Fig. 4, the four KM survival curves were for each of the four groups based on the median of the LAVi and LA reservoir strain. When the atrium was not yet enlarged, a reduced LA strain had a poor prognosis. However, when strain function was normal, the atrial enlargement didn’t affect the prognosis. Furthermore, in the case of atrial enlargement, reservoir strain can further stratify the prognosis of the patient. Two patients who corroborated this finding are presented in Fig. 2.

### LA reservoir strain adds incremental value to clinical risk factors

**Fig. 5:**
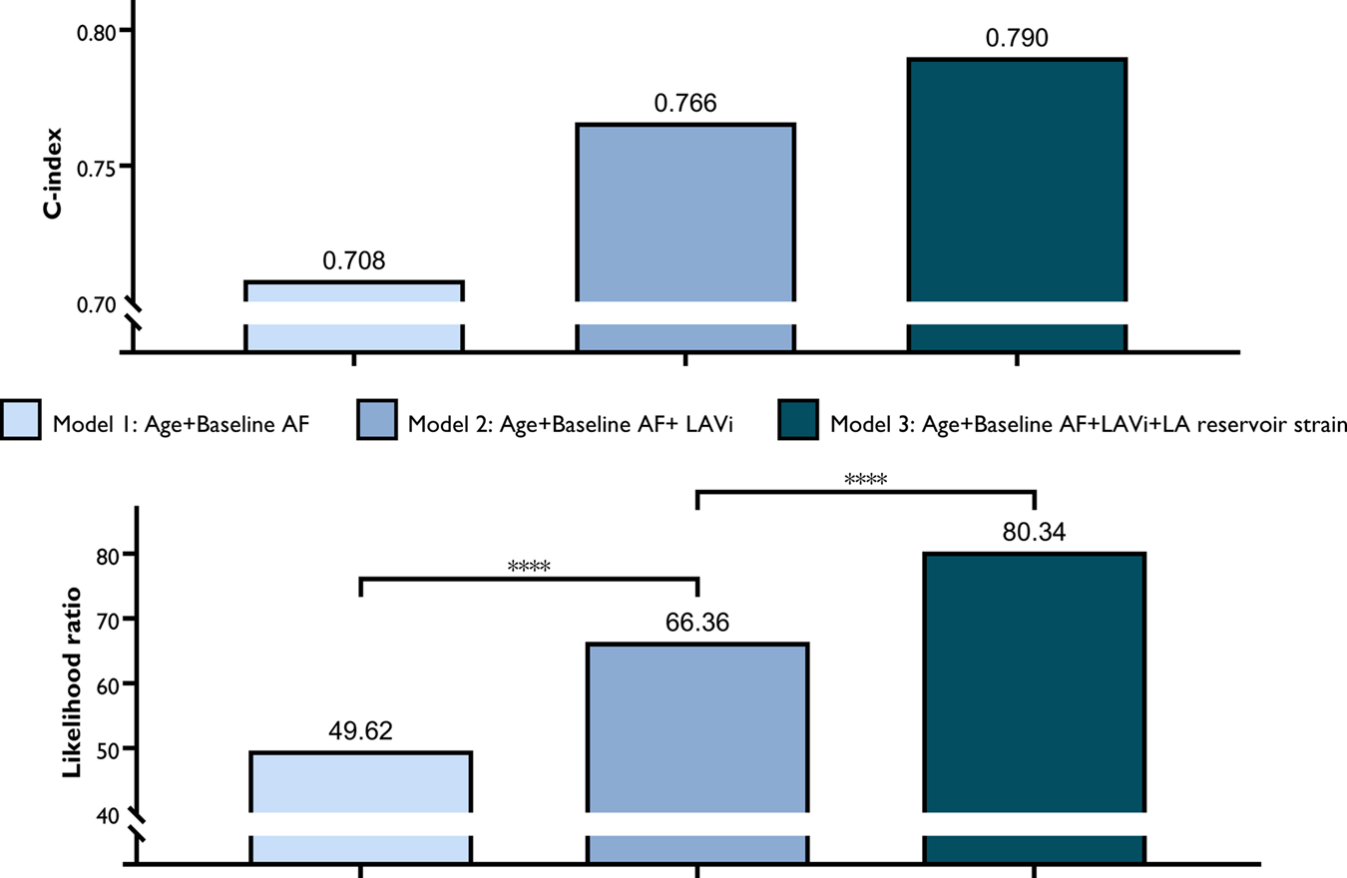
Incremental value of LA reservoir strain median with Harrell’s C-index and log-likelihood ratio test. AF, atrial fibrillation; LA, left atrial; LAVi, left atrial volume index. **** indicating *p* < 0.0001

We evaluated the accuracy and calibration of LA reservoir strain by adding LAVi and reservoir strain to age and baseline AF as independent factors step-by-step (Fig. 5). The addition of the two indicators increased the C-index of the model from the original 0.708 to 0.766 and to 0.790 in order. Additionally, the likelihood ratio increased from 49.62 to 66.36 and to 80.34. There was a significant difference between the three models (*p* < 0.0001). Therefore, the LA reservoir strain demonstrated an incremental value in the prognosis of MACE in ApHCM patients. Similarly, the incremental value of the LA conduit and booster strain are displayed in Figs. S3 and S4.

### Intra- and inter-observer reproducibility

Table 4 reveals the reproducibility of the LA longitudinal strain. ICC of LA deformation parameters for intra-observer variability ranged between 0.89 (95% CI: 0.75–0.96) (booster strain) and 0.93 (95% CI: 0.82–0.97) (conduit strain), and for inter-observer variability ranged between 0.93 (95% CI: 0.83–0.97) (conduit strain) and 0.98 (95% CI: 0.94–0.99) (booster strain). LA reservoir strain maintained moderately stable intra- and inter-observer reproducibility, 0.92 (95% CI: 0.80–0.97) and 0.95 (95% CI: 0.87–0.98), respectively.

**Table 4:** The inter- and intra-observer variability of left atrial longitudinal strain

LA strain	Inter-observer		Intra-observer	
	ICC	95% CI	ICC	95% CI
Reservoir	0.95	0.87–0.98	0.92	0.80–0.97
Conduit	0.93	0.83–0.97	0.93	0.82–0.97
Booster	0.98	0.94–0.99	0.89	0.75–0.96

*LA* left atrial, *ICC* intra-class correlation coefficient, *CI* confidence interval

### Subgroup analysis

**Fig. 6:**
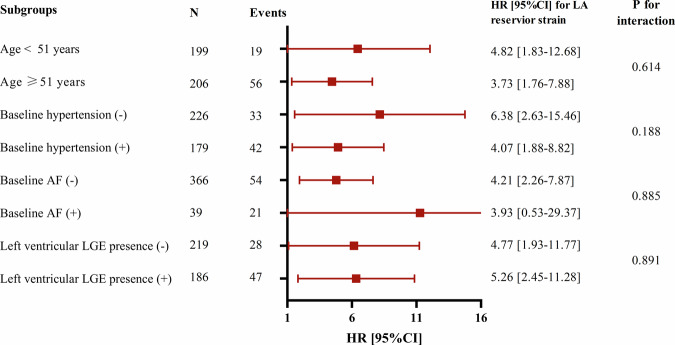
Hazard ratio for left atrial reservoir strain in association with adverse outcome in various subgroups. AF, atrial fibrillation; LGE, late gadolinium enhancement; HR, hazard ratio

In subgroups stratified by age, presence of baseline hypertension or not, presence of baseline AF or not, and presence of LV LGE or not, the interaction failed to reach statistical significance in the association of decreased LA reservoir strain with MACE (all *p*-value > 0.05) (Fig. 6).

## Discussion

In this study, we recognized that in addition to baseline data of age and AF, increased LA volume and decreased LA longitudinal strain by CMR are highly correlated with prognosis in patients with ApHCM. Importantly, the LA reservoir function was found to be an independent predictor of MACE and an incremental value to the clinical risk factors and LAVi. Routine measurement of LA strain parameters in CMR cine sequences may be useful for clinical risk stratification and further treatment of patients.

To date, our study is one of the largest samples, and it provides new valuable insights into the prognosis of ApHCM. More than 40 years after first described in Japan, ApHCM has not received much attention and has not been fully studied, compared to classic HCM (asymmetric hypertrophy of the interventricular septum) [31, 32]. In a U.S. population-based cohort study, *n* = 55 of 187 patients (29.4%) died, including 7 cardiac deaths [3]. However, in other Asian cohorts, patients with ApHCM did not experience SCD or HF [33, 34]. In our cohort, SCD-related events occurred in 14 participants; these included 6 SCDs and 8 aborted SCDs. Seven other patients died of other causes. Fifteen participants experienced HF-related events, including 2 heart transplants and 13 hospitalizations for HF.

Increasing age and LAVi have long been shown to be highly correlated with the prognosis of patients with ApHCM [3, 35]. AF, a common type of supraventricular arrhythmia, is inextricably linked to the occurrence of MACE events in patients with HCM [12]. The importance of AF has been mentioned in several ApHCM cohorts and even more so in our prognostic outcome [19, 36–38]. It is worth mentioning that the causal relationship between AF and LA enlargement is still an incompletely resolved issue [39, 40]. In this study, LV outflow tract obstruction, degree of hypertrophy, and LV LGE were not as significant as in previous studies [9]. We speculate that this may be related to the fact that patients with ApHCM have less hypertrophy than normal HCM, and late fibrosis is not yet evident. To this end, we supplemented the KM curves with LGE grouped according to cut-off values.

In recent years, LA deformation has been recognized as a valuable prognostic marker, surpassing LA EF and LAVi [16–18, 30]. Previous studies have demonstrated the prognostic significance of LA enlargement in HCM patients, and LA diameter has been included in the SCD risk prediction model for patients with HCM [41]. In a large study by Nistri et al that included 1491 patients with HCM in Italy, the independent predictive value of the LA dimension was confirmed, particularly in identifying patients at risk of HF-related mortality [14]. In the study by Hiemstra et al, patients with an LAVi less than a cut-off of 34 mL/m^2^ had better survival rates [13]. However, there was only one CMR-based study that assessed LA strain in ApHCM [42]. A small study including 15 ApHCM patients by Kao et al discussed the high correlation between impaired LA conduit strain measured by echocardiography and the development of non-valvular AF [43]. Notably, in our results, the LA strain was linked to the prognosis of ApHCM on CMR for the first time. The LA reservoir function, as a sensitive surrogate for LA mechanical dysfunction and LV diastolic function, significantly predicted patient outcomes and was more sensitive than volumetric indicators [11, 44]. LA dysfunction may precede LA enlargement and is a promising metric to assess clinical implications and predict prognosis at the early stage. However, the enlargement of the atrium had no prognostic value when the atrial strain was not reduced.

CMR is routinely recommended for risk stratification in HCM patients and its feature-tracking technique is accessible in cine sequences [22, 30, 45]. Under normal physiological conditions, active atrial contraction contributes only about 25% to ventricular filling during the late diastolic phase of LV. When LV diastolic dysfunction occurs, the atrium exhibits complex phasic function and modulates for the lack of filling in early diastole by compensatory work to maintain cardiac output. Prolonged overload of the LA volume will lead to atrial enlargement and atrial myocardial fibrosis, which in turn leads to compensatory hypertrophy and enlargement of the atrial, also known as LA remodeling. In the long term, LA function decreases, increasing the risk of MACE. LA function can be assessed by the strain. LA long-axis strain can be divided into three phases: reservoir, conduit, and booster, which are sensitive, reproducible, and easily obtained by CMR. Decreased LA reservoir, conduit, and booster strain have been shown to be associated with MACE in HCM patients in several CMR studies [11, 17, 18, 46, 47]. However, given the varying endpoint events and inclusion criteria, not exactly the same conclusions were drawn about the predictive power of LA three-phase strain. In our study focused on ApHCM, the reservoir strain had the best prognostic value and incremental model prediction performance in the LA three-phase strain. When included as categorical variables in the multivariable Cox regression model, all three phases of LA strain were independently associated with the outcomes. This finding reflects the stability of the LA strain measured by MRI. However, when we dropped them into the model as continuous variables, the conduit strain lost its ability to prognosis. This is an area that requires further research. Additionally, LA strain is highly related to LV strain and LV/LA volume, and strain rate was not involved in this study. This is an important consideration for future research.

### Limitation

Firstly, this was a large retrospective study on ApHCM performed in a tertiary referral center, thus selection and referral bias may be present. Secondly, the number of patients with all-cause mortality and heart transplants in our study was limited. Therefore, the study endpoints in this study were composite endpoints, which also makes our results exploratory, requiring validation in larger cohorts and longer follow-up for more hard clinical endpoints. The prognostic value of the LA reservoir strain was consistent across subgroups, but the subgroup analyses had limited sample sizes and the number of events.

## Conclusions

In conclusion, our study demonstrates that in addition to age and previous history of AF, increased LA volume and decreased LA longitudinal strain by CMR are highly correlated with prognosis in patients with ApHCM. LA reservoir strain has potential incremental prognostic value.

## Supplementary information

ELECTRONIC SUPPLEMENTARY MATERIAL

## Abbreviations

AF: Atrial fibrillation
ApHCM: Apical hypertrophic cardiomyopathy
AUC: Area under the curve
CMR: Cardiac magnetic resonance
EF: Ejection fraction
HCM: Hypertrophic cardiomyopathy
HF: Heart failure
HR: Hazard ratio
ICC: Intra-class correlation coefficient
IQR: Interquartile range
KM: Kaplan–Meier
LA: Left atrial/atrium
LAVi: Left atrial volume index
LGE: Late gadolinium enhancement
LV: Left ventricular/ventricle
MACE: Major adverse cardiovascular events
MRI: Magnetic resonance imaging
NYHA: New York Heart Association
ROC: Receiver operating characteristic
ROI: Region of interest
SCD: Sudden cardiac death
