# Supplementary material for: Incremental prognostic value of left atrial strain in apical hypertrophic cardiomyopathy: a cardiovascular magnetic resonance study
Source: Eur Radiol. 2024 Sep 18;35(4):1818–29. doi: 10.1007/s00330-024-11058-y (PMC11914350; doi:10.1007/s00330-024-11058-y)
Supplement: Supplementary file 1 — ELECTRONIC SUPPLEMENTARY MATERIAL [file 330_2024_11058_MOESM1_ESM.pdf]

Supplemental Figures and Figure Legends

Figure S1. Kaplan-Meier survival curves for subgroups divided by LV LGE extent cut-off 1.33%

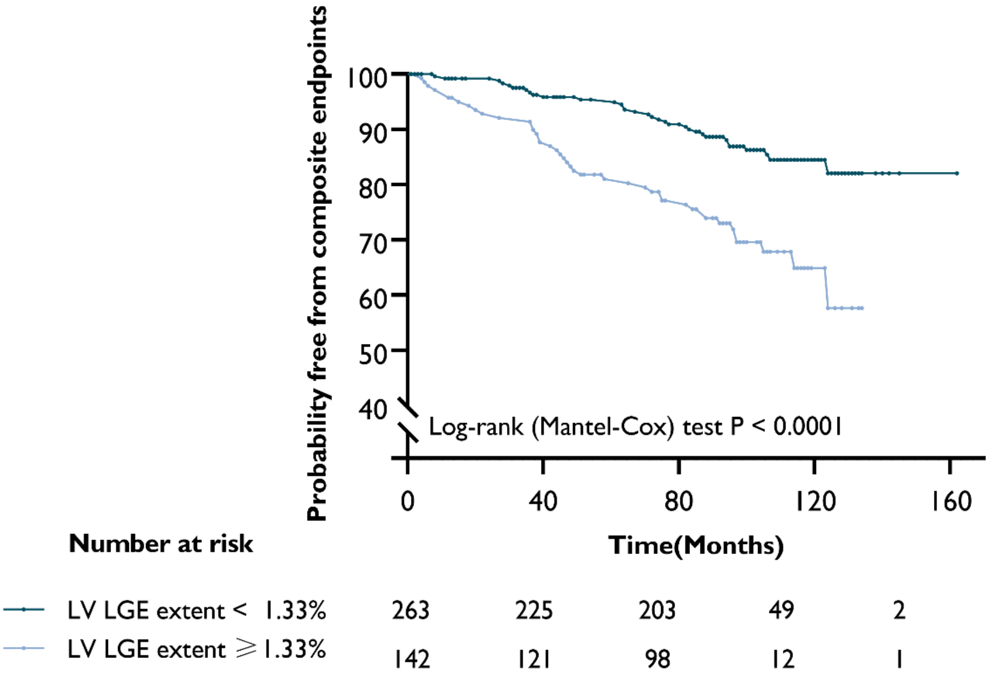

LV, left ventricular; LGE, late gadolinium enhancement.

Figure S2. Receiver operating characteristic curves of LA parameters

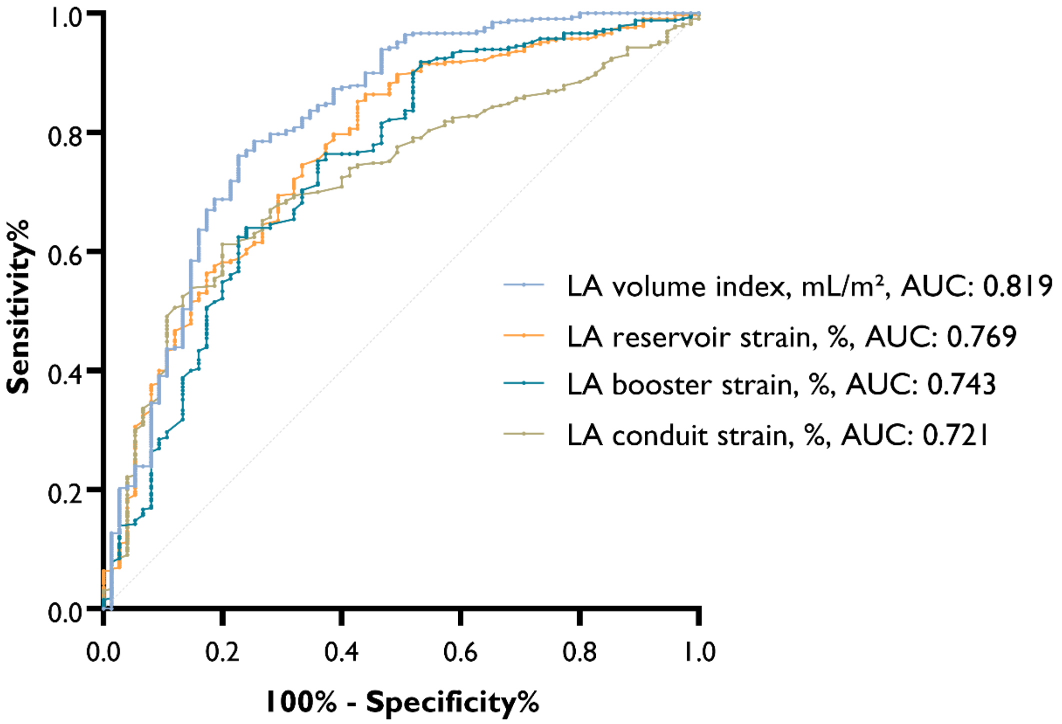

LA, left atrial.

Figure S3. Incremental value of LA conduit strain median with Harrell's C-index and log likelihood ratio test

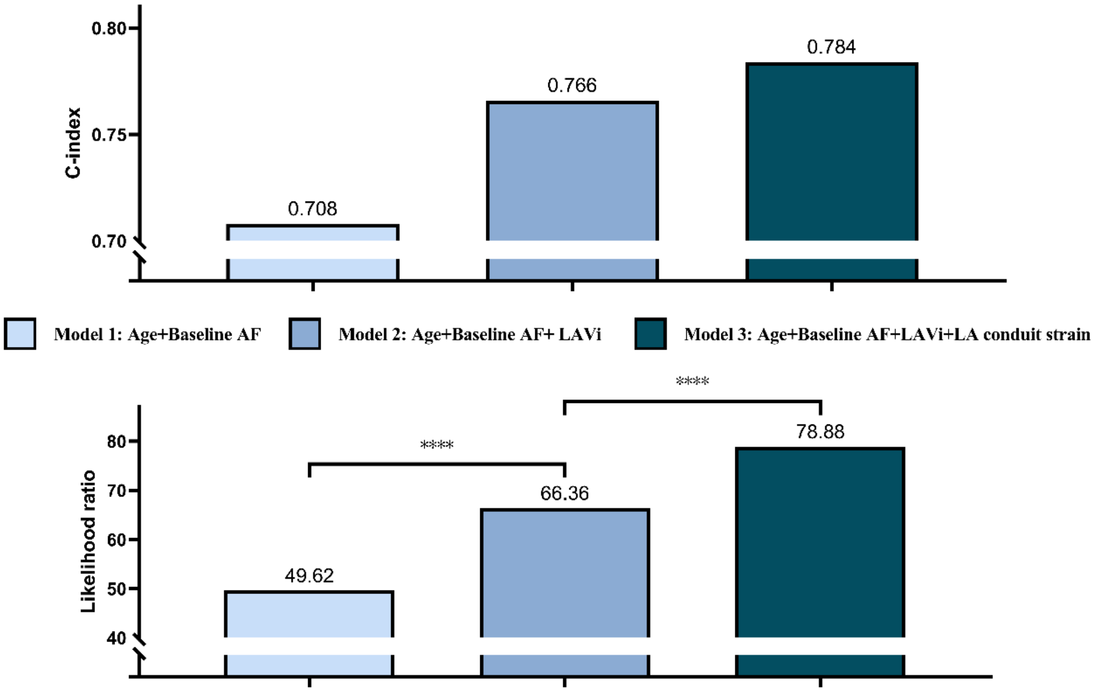

AF, atrial fibrillation; LA, left atrial; LAVi, left atrial volume index.

Figure S4. Incremental value of LA booster strain median with Harrell's C-index and log likelihood ratio test

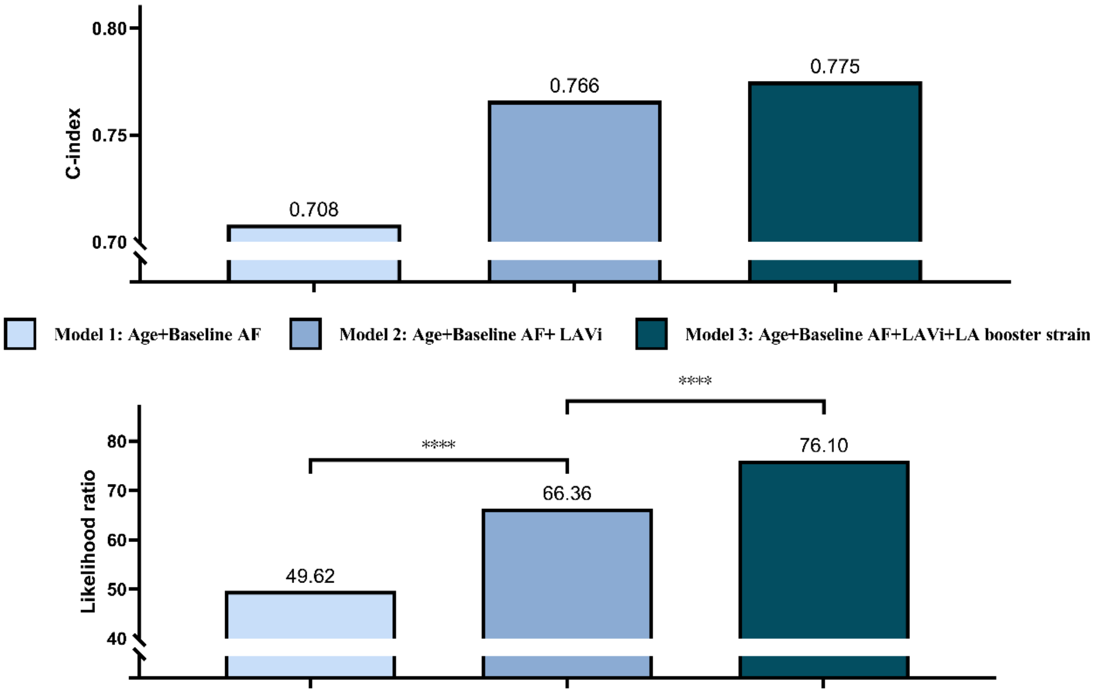

AF, atrial fibrillation; LA, left atrial; LAVi, left atrial volume index.

Supplemental Tables

Table S1. Univariable predictors of cardiovascular endpoint in all patients

**Univariable Unadjusted**	**HR [95%-CI]**	**P-value**
Male, %	0.75 [0.44, 1.27]	0.279
Age, yrs	1.06 [1.04, 1.08]	**<0.001**
Age ≥ 51 years	3.40 [2.02, 5.73]	**<0.001**
BMI, kg/m^2	0.97 [0.90, 1.05]	0.444
History of hypertension, %	1.79 [1.14, 2.83]	**0.012**
NYHA class III/IV, %	2.77 [1.27, 6.03]	**0.011**
Family history of HCM, %	1.16 [0.59, 2.25]	0.668
History of atrial fibrillation, %	6.13 [3.67, 10.23]	**<0.001**
Non-sustained ventricular tachycardia, %	2.97 [0.41, 21.38]	0.281
**Left ventricular parameters**		
Maximal wall thickness, mm	1.10 [1.05, 1.15]	**<0.001**
Maximal wall thickness ≥ 15mm	3.07 [1.74, 5.41]	**<0.001**
Ejection fraction, %	1.01 [0.98, 1.04]	0.467
Apical aneurysm, %	3.11 [0.98, 9.89]	0.054
End-diastolic volume index, mL/m^2	1.00 [0.98, 1.01]	0.695
End-systolic volume index, mL/m^2	1.00 [0.96, 1.04]	0.982
Stroke volume index, mL/m^2	0.99 [0.97, 1.02]	0.573
Cardiac index, L/min/m^2	0.86 [0.61, 1.20]	0.378
Mass index, g/m^2	1.01 [1.00, 1.02]	0.120
LGE presence, %	2.16 [1.35, 3.45]	**0.001**
LGE extent, %	1.09 [1.05, 1.13]	**<0.001**
Mid-LV obliteration presence, %	1.18 [0.15, 9.15]	0.873
**Left atrial parameters**		
Anteroposterior diameter, mm	1.11 [1.08, 1.15]	**<0.001**
Volume index, mL/m^2	1.04 [1.03, 1.06]	**<0.001**
Volume index ≥ 38.77mL/m^2	3.26 [1.93, 5.48]	**<0.001**
Ejection fraction, %	0.95 [0.94, 0.96]	**<0.001**
Ejection fraction≤54.20%	3.90 [2.29, 6.62]	**<0.001**
Reservoir strain, %	0.91 [0.89, 0.93]	**<0.001**
Reservoir strain≤29.4%	5.26 [2.94, 9.40]	**<0.001**
Conduit strain, %	0.87 [0.83, 0.91]	**<0.001**
Conduit strain≤11.6%	4.85 [2.75, 8.55]	**<0.001**
Booster strain, %	0.87 [0.85, 0.90]	**<0.001**
Booster strain≤17.4%	4.51 [2.59, 7.83]	**<0.001**

HR, hazard ratio; BMI, body mass index; NYHA, New York Heart Association; LGE, late gadolinium enhancement.
